# Serum progranulin as a predictive marker for high activity of antineutrophil cytoplasmic antibody‐associated vasculitis

**DOI:** 10.1002/jcla.24048

**Published:** 2021-10-09

**Authors:** Taejun Yoon, Lucy Eunju Lee, Sung Soo Ahn, Jung Yoon Pyo, Jason Jungsik Song, Yong‐Beom Park, Sang‐Won Lee

**Affiliations:** ^1^ Department of Medical Science College of Medicine BK21 Plus Project Yonsei University Seoul Republic of Korea; ^2^ Division of Rheumatology Department of Internal Medicine Yonsei University College of Medicine Seoul Republic of Korea; ^3^ Institute for Immunology and Immunological Diseases Yonsei University College of Medicine Seoul Republic of Korea

**Keywords:** antineutrophil cytoplasmic antibody, Birmingham vasculitis activity score, progranulin, vasculitis

## Abstract

**Background:**

This study investigated whether serum progranulin could act as a predictive marker for high disease activity of antineutrophil cytoplasmic antibody (ANCA)‐associated vasculitis (AAV).

**Methods:**

Fifty‐eight AAV patients were included in this study. Clinical and laboratory data were obtained at blood collection. The Short‐Form 36‐Item Health Survey Physical and Mental Component Summaries (SF‐36 PCS and SF‐36 MCS), Birmingham Vasculitis activity score (BVAS), Five‐Factor Score (FFS), and Vasculitis Damage Index (VDI) were assessed as AAV‐specific indices. Whole blood was collected and serum samples were isolated and stored at −80°C. Serum progranulin concentration was quantified by ELISA kits.

**Results:**

The median age of patients was 63.0 years (19 men). The median BVAS was 11.0, and the median serum progranulin level was 49.0 ng/ml. Serum progranulin was significantly correlated with BVAS, FFS, erythrocyte sedimentation rate, C‐reactive protein level, SF‐36 PCS, haemoglobin, and serum albumin. Severe AAV was arbitrarily defined as the highest tertile of BVAS (BVAS ≥16). When the cut‐offs of serum progranulin were set as 55.16 ng/ml and 43.01 ng/ml for severe AAV, AAV patients with serum progranulin ≥55.16 and 43.01 ng/ml had significantly higher risks of severe AAV than those without (relative risk (RR) 4.167 and 4.524, respectively).

**Conclusions:**

Progranulin might play an anti‐inflammatory role in AAV pathogenesis and serum progranulin could be used as a predictive marker for high activity of AAV.

## Graphical abstract

Serum progranulin was significantly correlated with BVAS (*r* = 0.342) as well as acute‐phase reactants such as ESR (*r* = 0.379) in AAV patients. AAV, antineutrophil cytoplasmic antibody‐associated vasculitis; BVAS, Birmingham vasculitis activity score; CRP, C‐reactive protein; ESR, erythrocyte sedimentation rate.

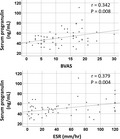

## 1 INTRODUCTION

Progranulin, a 68.5 kDa protein consisting of 593 amino acids, is known to participate in cell development, cell proliferation, and wound healing.^1^ Progranulin is a precursor for several types of granulins and is cleaved into granulins by elastase, proteinase 3, and matrix metalloproteinase present in the extracellular matrix and lysosomes.^2^ Progranulin is known to assume both pro‐inflammatory roles and anti‐inflammatory roles in the immune response. Binding of progranulin to ephrin type‐A receptor 2 could trigger its pro‐inflammatory effect by accelerating inflammation and upregulating progranulin expression through the mitogen‐activated protein kinase and protein kinase B pathways.^3^ Additionally, progranulin augments and strengthens toll‐like receptor (TLR 9)‐mediated intracellular signalling, which may play a pro‐inflammatory role.^3^ Conversely, progranulin may exert an anti‐inflammatory effect by binding to tumour necrosis factor (TNF) receptor (TNFR)1 and TNFR2 with an affinity comparable to that to TNF‐α, resulting in inhibition of TNF‐α binding to its receptor.^4^ Furthermore, progranulin may polarise Treg and enhance IL‐10 production.^5^ The role of progranulin in the inflammatory process of several autoimmune diseases remains controversial: its pro‐inflammatory action was reported to exhibit a significant correlation with granulin levels and disease activity in systemic lupus erythematosus (SLE) and systemic sclerosis, whereas its anti‐inflammatory action was known to be implicated in rheumatoid arthritis (RA), inflammatory bowel disease, and psoriasis.^4^, ^6^

An array of immune cells and pro‐inflammatory cytokines are known to participate in the pathogenesis of antineutrophil cytoplasmic antibody (ANCA)‐associated vasculitis (AAV).^7^, ^8^ Therefore, progranulin may contribute to inflammation or reflect the inflammatory burden in AAV patients. However, there is no study to date investigating the clinical significance of progranulin in AAV patients. In this context, we investigated here whether serum progranulin could act as a predictive marker for high disease activity in AAV patients.

## 2 PATIENTS AND METHODS

### 2.1 Study subjects

We selected 58 AAV patients with consecutive hospital identity numbers from the Severance Hospital ANCA‐associated VasculitidEs (SHAVE) cohort. The SHAVE cohort, established in November 2016, is a prospective and observational cohort including patients with microscopic polyangiitis (MPA), granulomatosis with polyangiitis (GPA), and eosinophilic granulomatosis with polyangiitis (EGPA). These patients were first diagnosed with AAV at our hospital between January 2019 and December 2020. All AAV patients in the SHAVE cohort met both the 2007 European Medicines Agency algorithms for AAV and polyarteritis nodosa and the 2012 revised International Chapel Hill Consensus Conference Nomenclature of Vasculitides.^9^, ^10^ This study was approved by the Institutional Review Board (IRB) of Severance Hospital (4‐2016‐0901) and, when required, written informed consent was obtained from patients at the time of collection of blood samples. The IRB waived the need for written informed consent when it had been previously obtained during patient enrolment in the SHAVE cohort. This study was in compliance with the Declaration of Helsinki.

### 2.2 Collection of clinical and laboratory data and AAV‐specific indices

Data regarding age, sex, AAV subtypes, ANCAs, and organ involvement were collected from patients during the visit for blood sample collection. As laboratory data, white blood cell and platelet counts, erythrocyte sedimentation rate (ESR), C‐reactive protein (CRP), haemoglobin, glucose, blood urea nitrogen, serum creatinine, total protein, serum albumin, and complements 3 and 4 were collected. The Korean version of the Short‐Form 36‐item health survey physical and mental component summaries (SF‐36 PCS and SF‐36 MCS),^11^ Birmingham vasculitis activity score (BVAS, version 3),^12^ five‐factor score (FFS),^13^ and vasculitis damage index (VDI)^14^ were assessed as AAV‐specific indices.

### 2.3 Blood collection and storage

Whole blood samples were collected after patients’ consent and serum samples were isolated and stored at −80°C. Clinical and laboratory data and AAV‐specific indices were obtained on the same day as that for blood sample collection.

### 2.4 Estimation of serum progranulin

Serum progranulin concentration was quantified by ELISA kits (R&D Systems) from stored sera according to the manufacturer's instruction.

### 2.5 Statistical analyses

All statistical analyses were performed using IBM SPSS Statistics for Windows, version 25 (IBM Corp.). Continuous and categorical variables are expressed as medians with interquartile ranges and numbers (percentages), respectively. The Mann‐Whitney U test was used to check for significant differences between two groups on continuous variables. The correlation coefficient (r) between two variables was obtained using either the Pearson's correlation analysis or univariable linear regression analysis. Multivariable linear regression analysis was performed using statistically significant variables identified from the univariable analysis. The odds ratio (OR) was obtained using multivariable logistic regression analysis of variables with *p* < 0.005 in the univariable logistic regression analysis. The optimal cut‐off was extrapolated by performing receiver operator characteristic (ROC) curve analysis, and the maximum sum of sensitivity and specificity was selected. The relative risk (RR) of the cut‐off for high disease activity of AAV was analysed using contingency tables and the chi‐square test. *p*‐values less than 0.05 were considered statistically significant.

## 3 RESULTS

### 3.1 Characteristics of patients

Clinical and laboratory data and the AAV‐specific indices estimated at the time of blood collection are shown in Table 1. The median age of patients was 63.0 years, and 19 of the patients were men. A total of 29 patients were diagnosed with MPA, 17 with GPA, and 12 with EGPA. The median BVAS was 11.0, and the median serum progranulin was 49.0 ng/ml. Glucocorticoids and azathioprine were being administered to 44 and 17 of the patients, respectively, at the time of blood collection (Table 1).

**TABLE 1:** Characteristics of 58 patients with AAV

Variables at the time of blood collection	Values
Demographic data	
Age (years)	63.0 (21.0)
Male sex, *N* (%)	19 (32.8)
AAV subtypes, *N* (%)	
MPA	29 (50.0)
GPA	17 (29.3)
EGPA	12 (20.7)
ANCA positivity, *N* (%)	
MPO‐ANCA(or P‐ANCA) positive	39 (67.2)
PR3‐ANCA (or C‐ANCA) positive	8 (13.8)
Both ANCAs	1 (1.7)
ANCA negative	12 (20.7)
AAV‐specific indices	
SF‐36 PCS	50.6 (35.8)
SF‐36 MCS	54.2 (36.4)
BVAS	11.0 (11.0)
FFS	0 (2.0)
VDI	3.0 (2.0)
Organ involvement, *N* (%)	
General symptoms	25 (43.1)
Skin	9 (15.5)
Mucosa and eyes	2 (3.4)
Ear nose and throat	25 (43.1)
Lungs	42 (72.4)
Heart	5 (8.6)
Gastrointestine	2 (3.4)
Kidneys	32 (55.2)
Central and peripheral nervous systems	20 (34.5)
Acute phase reactants	
ESR (mm/h)	34.0 (75.0)
CRP (mg/L)	5.3 (43.2)
Laboratory results	
White blood cell count (/mm^3^)	8,330.0 (7270.0)
Neutrophil (/mm^3^)	5,785.0 (6110.0)
Lymphocyte (/mm^3^)	1,430.0 (910.0)
Haemoglobin (g/dl)	11.0 (4.0)
Platelet count (x1,000/mm^3^)	263.0 (142.0)
Glucose (mg/dl)	105.0 (41.0)
Blood urea nitrogen (mg/dl)	20.5 (24.1)
Serum creatinine (mg/dl)	0.9 (1.7)
Total protein (g/dl)	6.5 (0.8)
Serum albumin (g/dl)	3.7 (1.1)
Complement 3 (mg/dl)	113.2 (37.9)
Complement 4 (mg/dl)	25.6 (12.1)
Serum Progranulin (ng/ml)	49.0 (17.9)
At the time of blood collection	
Immunosuppressive drugs administered, *N* (%)	
Glucocorticoids	44 (75.9)
Cyclophosphamide	5 (8.6)
Rituximab	2 (3.4)
Azathioprine	17 (29.3)
Mycophenolate mofetil	0 (0)
Tacrolimus	0 (0)
Methotrexate	1 (1.7)

Note

Values are expressed as median (interquartile range [IQR]) or number (percentage).

Abbreviations: AAV, ANCA‐associated vasculitis; ANCA, antineutrophil cytoplasmic antibody; BVAS, Birmingham vasculitis activity score; C, cytoplasmic; CRP, C‐reactive protein; EGPA, eosinophilic granulomatosis with polyangiitis; ESR, erythrocyte sedimentation rate; FFS, five‐factor score; GPA, granulomatosis with polyangiitis; MCS, mental component summary; MPA, microscopic polyangiitis; MPO, myeloperoxidase; P, perinuclear; PCS, physical component summary; PR3, proteinase 3; SF‐36, short‐form 36‐item; VDI, vasculitis damage index.

### 3.2 Correlation analysis

**FIGURE 1:**
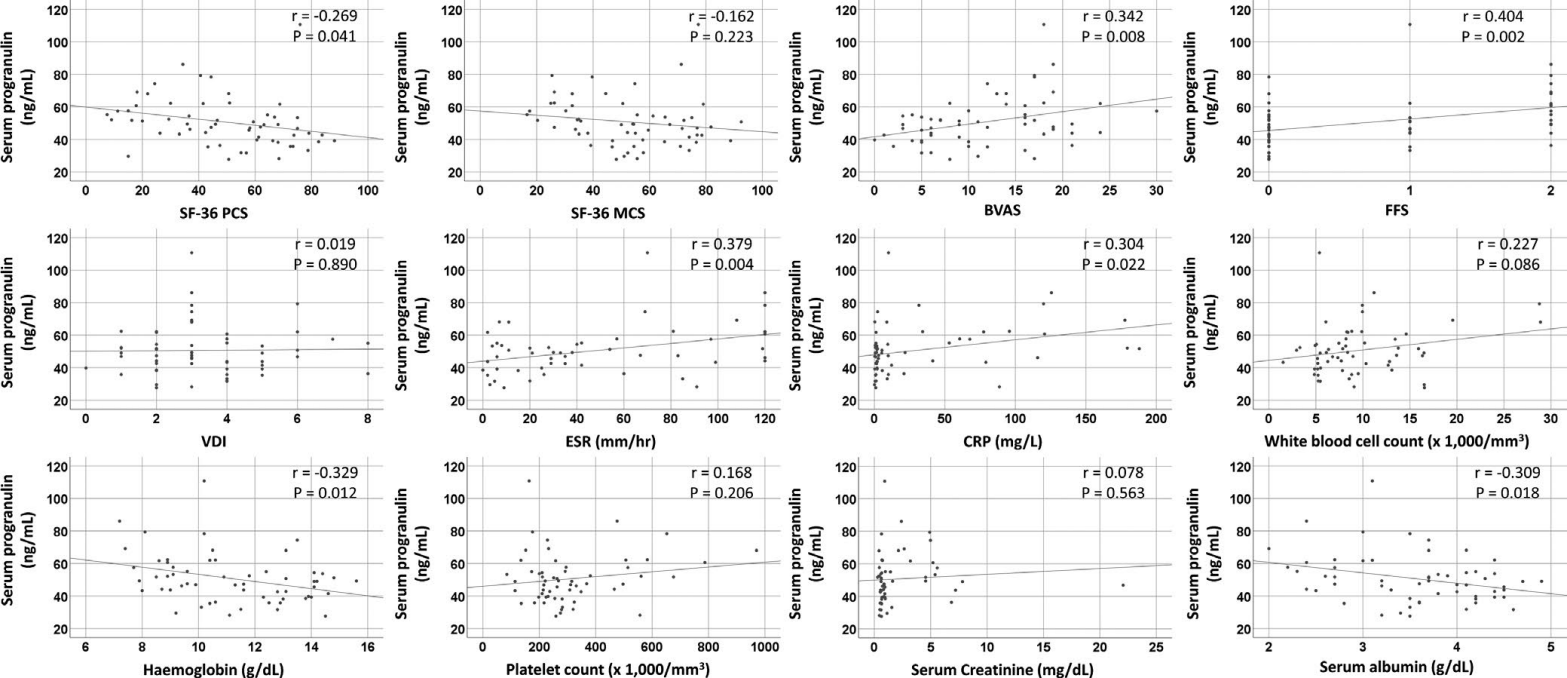
Serum progranulin was positively correlated with BVAS, FFS, ESR, and CRP level, whereas it was negatively correlated with SF‐36 PCS, haemoglobin, and serum albumin. BVAS, Birmingham Vasculitis activity score; CRP, C‐reactive protein; ESR, erythrocyte sedimentation rate; FFS, five‐factor score; SF‐36 PCS, the Short‐Form 36‐item health survey physical component summaries

Serum progranulin was positively correlated with BVAS (*r* = 0.342), FFS (*r* = 0.404), ESR (*r* = 0.379), and CRP level (*r* = 0.304), whereas, it was negatively correlated with SF‐36 PCS (*r* = −0.269), haemoglobin (*r* = −0.329), and serum albumin (*r* = −0.309) (Figure 1). Among the nine organ involvements explored, serum progranulin was significantly correlated with the total score of general symptoms (*r* = 0.286, *p* = 0.029) and that of kidney involvement (*r* = 0.387, *p* = 0.003) (Figure S1A). In addition, AAV patients with general symptoms exhibited a significantly higher median serum progranulin than those without such symptoms (52.1 vs. 44.2 ng/ml, *p* = 0.014). However, the median serum progranulin did not show significant differences (*p* = 0.104) between patients with and without kidney involvement (Figure S1B).

### 3.3 Cut‐off value of serum progranulin for the cross‐sectional severe AAV

**FIGURE 2:**
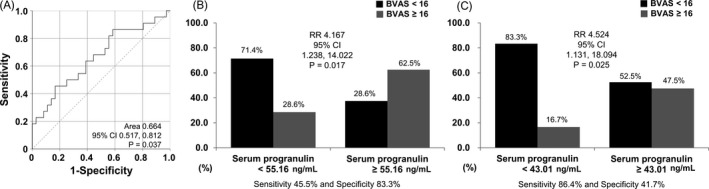
A) When the cut‐offs of serum progranulin were set as 55.16 ng/ml and 43.01 ng/ml for severe AAV, the sensitivity and specificity were 45.5% and 83.3%, and 86.4% and 41.7%, respectively. B) When the cut‐off of serum progranulin was set as 55.16 ng/ml, serum progranulin ≥55.16 ng/ml had a significantly higher risk of severe AAV than those with serum progranulin <55.16 ng/ml (RR 4.167). C) When the cut‐off of serum progranulin was set as 43.01 ng/ml, serum progranulin ≥43.01 ng/ml had a significantly higher risk of severe AAV than those with serum progranulin <43.01 ng/ml (RR 4.524). AAV, antineutrophil cytoplasmic antibody‐associated vasculitis; RR, relative risk

Severe AAV was defined as the highest tertile of BVAS (BVAS ≥16) estimated in this study. The optimal cut‐off value of serum progranulin for severe AAV was obtained using the ROC curve (area 0.664, 95% confidence interval (CI) 0.517, 0.812, *p* = 0.037). When the cut‐off was set at 55.16 ng/ml, the sensitivity and specificity were 45.5% and 83.3%, respectively. However, when the cut‐off was set at 43.01 ng/ml, the sensitivity increased up to 86.4% whereas the specificity decreased to 41.7% (Figure 2A).

### 3.4 Estimation of relative risk

When AAV patients were divided into two groups based on a cut‐off serum progranulin of 55.16 ng/ml, those with serum progranulin ≥55.16 ng/ml had a significantly higher risk of severe AAV than those with serum progranulin <55.16 ng/ml (RR 4.167, 95% CI 1.238, 14.022) (Figure 2B). A similar trend was observed for AAV patients assigned into two groups according to the cut‐off of 43.01 ng/ml of serum progranulin (RR 4.524, 95% CI 1.131, 18.094) (Figure 2C).

### 3.5 Linear regression analysis for the cross‐sectional BVAS

The variables estimated at the time of blood collection for the cross‐sectional BVAS were subjected to a univariate analysis. SF‐36 PCS, SF‐36 MCS, FFS, VDI, ESR, CRP, white blood cell and platelet counts, haemoglobin, glucose, blood urea nitrogen, serum albumin, and serum progranulin were all significantly correlated with the cross‐sectional BVAS. In the multivariable analysis, only FFS, VDI, ESR, CRP, haemoglobin, and serum albumin were identified to be significantly associated with the cross‐sectional BVAS. However, serum progranulin was not independently associated with the cross‐sectional BVAS (Table 2).

**TABLE 2:** Linear regression analysis of continuous variables for simultaneous BVAS

Variables	Univariable			Multivariable		
	Beta	95% CI	*p* value	Beta	95% CI	*p* value
Age	0.123	−0.066, 0.180	0.357			
SF‐36 PCS	−0.428	−0.207, −0.057	0.001	0.054	−0.076, 0.110	0.714
SF‐36 MCS	−0.393	−0.222, −0.051	0.002	−0.010	−0.095, 0.087	0.937
FFS	0.519	2.270, 5.841	<0.001	0.314	0.492, 4.619	0.017
VDI	0.293	0.142, 2.125	0.026	0.180	0.033, 1.395	0.040
ESR	0.602	0.063, 0.136	<0.001	0.390	0.027, 0.103	0.001
CRP	0.449	0.028, 0.093	<0.001	−0.314	−0.077, −0.010	0.013
White blood cell count	0.282	0.031, 0.677	0.032	0.124	−0.119, 0.473	0.235
Haemoglobin	−0.669	−2.583, −1.400	<0.001	−0.320	−1.726, −0.220	0.013
Platelet count	0.262	0.000, 0.000	0.047	0.072	−0.006, 0.012	0.539
Glucose	0.285	0.005, 0.101	0.030	0.131	−0.010, 0.066	0.138
Blood urea nitrogen	0.322	0.023, 0.193	0.014	−0.076	−0.109, 0.055	0.511
Serum creatinine	0.020	−0.58, 0.589	0.883			
Total protein	−0.227	−4.360, 0.303	0.087			
Serum albumin	−0.681	−8.109, −4.488	<0.001	−0.306	−5.479, −0.185	0.037
Complement 3	−0.202	−0.094, 0.015	0.151			
Complement 4	−0.041	−0.179, 0.134	0.770			
Serum progranulin	0.342	0.040, 0.264	0.008	−0.017	−0.091, 0.075	0.847

Abbreviations

BVAS, Birmingham vasculitis activity score; CRP, C‐reactive protein; ESR, erythrocyte sedimentation rate; FFS, five‐factor score; MCS, mental component summary; PCS, physical component summary; SF‐36, short‐form 36‐item; VDI, vasculitis damage index.

### 3.6 Logistic regression analysis for the cross‐sectional severe AAV

Two cut‐off values of serum progranulin, 55.16 and 43.01 ng/ml, were used in the logistic regression analysis. In the univariable analysis, SF‐36 PCS, SF‐36 MCS, FFS, ESR, CRP, haemoglobin, and serum albumin were found to be significantly associated with the cross‐sectional severe AAV. Serum progranulin higher than both 55.16 (OR 4.167) and 43.01 (OR 4.524) ng/ml were significantly associated with the cross‐sectional severe AAV. Multivariable analysis of data from patients with serum progranulin higher than 55.16 ng/ml identified FFS (OR 27.127, 95% CI 1.254, 586.817) and ESR (OR 1.091, 95% CI 1.013, 1.175) but not serum progranulin ≥55.16 ng/ml as independent predictors of the cross‐sectional severe AAV. Similarly, multivariable analysis of data from patients with serum progranulin higher than 43.01 ng/ml showed that FFS (OR 4.992, 95% CI 1.178, 21.152) and ESR (OR 1.042, 95% CI 1.010, 1.074) but not serum progranulin ≥43.01 ng/ml could predict the cross‐sectional severe AAV independently (Table 3).

**TABLE 3:** Logistic regression analysis of variables for simultaneous severe AAV

Variables	Univariable			Multivariable (Progranulin ≥55.16 ng/ml)			Multivariable (Progranulin ≥43.01 ng/ml)		
	OR	95% CI	*p* value	OR	95% CI	*p* value	OR	95% CI	*p* value
Age	1.012	0.975, 1.050	0.310						
SF‐36 PCS	0.976	0.948, 0999	0.040	1.027	0.949, 1.112	0.508	1.041	0.964, 1.124	0.308
SF‐36‐MCS	0.966	0.937, 0.996	0.025	1.035	0.944, 1.135	0.462	1.022	0.945, 1.105	0.583
FFS	2.701	1.382, 5.282	0.004	27.127	1.254, 586.817	0.035	4.992	1.178, 21.152	0.029
VDI	1.292	0.940, 1.775	0.114						
ESR	1.044	1.022, 1.066	<0.001	1.091	1.013, 1.175	0.022	1.042	1.010, 1.074	0.009
CRP	1.032	1.012, 1.052	0.002	1.009	0.978, 1.042	0.566	1.010	0.981, 1.039	0506
White blood cell count	1.075	0.970, 1.192	0.167						
Haemoglobin	0.418	0.270, 0.649	<0.001	0.396	0.149, 1.052	0.063	0.646	0.346, 1.204	0.169
Platelet count	1.003	0.999, 1.006	0.103						
Glucose	1.010	0.995, 1.026	0.184						
Blood urea nitrogen	1.021	0994, 1.048	0.132						
Serum creatinine	1.020	0.871, 1.195	0.807						
Total protein	0.643	0.309, 1.341	0.239						
Serum albumin	0.098	0.030, 0.318	<0.001	0.115	0.011, 1.226	0.073	0.213	0.026, 1.746	0.150
Complement 3	0.994	0.977, 1.010	0.444						
Complement 4	0.997	0.953, 1.043	0.897						
Serum progranulin ≥55.16 ng/ml	4.167	1.238, 14.022	0.021	0.008	0.000, 1.333	0.064			
Serum progranulin ≥43.01 ng/ml	4.524	1.131, 18.094	0.033				1.174	0.076, 18.052	0.909

Abbreviations

AAV, ANCA‐associated vasculitis; ANCA, antineutrophil cytoplasmic antibody; BVAS, Birmingham vasculitis activity score; C, cytoplasmic; CI, confidence interval; CRP, C‐reactive protein; ESR, erythrocyte sedimentation rate; Hb, haemoglobin; LUC, large unstained cell; MPO, myeloperoxidase; P, perinuclear; PLT, platelet; PR3, proteinase 3; WBC, white blood cell.

## 4 DISCUSSION

This study was designed to investigate the role of progranulin in AAV pathogenesis. In terms of a TLR9‐mediated pro‐inflammatory role, granulins, produced via the cleavage of progranulin by extracellular matrix proteases may increase the endocytosis of TLR9 ligands, thereby enhancing TLR9=associated intracellular signalling.^3^ This mechanism has been verified in SLE patients, in whom serum progranulin was shown to be significantly elevated when compared with that in healthy controls.^15^ TLR9 stimulation has also been reported to accelerate the activation, adhesion and degranulation of polymorphonuclear leucocytes (PMNs) in PR3‐ANCA vasculitis.^16^ Therefore, serum progranulin might bind to TLR9 with its ligands and thereby aggravate AAV by the activation and degranulation of PMNs. Conversely, the anti‐inflammatory effect of progranulin mediated by TNFR1 and TNFR2, leads to alleviation of TNF‐mediated inflammatory signalling by the competitive inhibition of TNF‐α binding to its receptors.^3^ This mechanism has been demonstrated in mice model for inflammatory arthritis; progranulin was found to bind to TNFRs and prevent inflammation by inhibiting TNF‐a‐activated intracellular signalling in animal models of inflammatory arthritis.^17^ Both TNFR1 and TNFR2 are known to be expressed at higher levels in AAV patients than in healthy controls, with the extents of their expression being significantly correlated with AAV activity based on BVAS.^18^ Therefore, the binding of serum progranulin to TNFR1 and TNFR2 could be speculated to improve AAV by competitive inhibition of TNF‐a binding to its receptors.

Therefore, consistent with the higher serum progranulin in SLE patients (in whom progranulin exhibited a pro‐inflammatory TLR‐mediated action) than in healthy controls, serum progranulin in RA patients (in whom progranulin assumes an anti‐inflammatory role through TNFR1 and TNFR2) was expected to be lower than that in healthy controls. However, serum progranulin in RA patients was also reported to be significantly higher than that in healthy controls.^19^ In this case, the increased serum progranulin was explained as an epiphenomenon that acts as negative feedback to alleviate inflammation,^20^ i.e. elevated serum progranulin may act as a direct pro‐inflammatory factor accelerating inflammation, but could also act as an indirect anti‐inflammatory factor for alleviating inflammation.

In the present study, serum progranulin was significantly correlated with BVAS in AAV patients. This is not consistent with the result of a previous study showing a positive correlation of serum progranulin with SLE activity based on the Systemic Lupus Erythematosus Disease Activity Index (SLEDAI) values. Progranulin is known to induce TLR9‐mediated inflammation in SLE patients^15^; therefore, this result could be explained by invoking the concept of negative feedback. As the activity of AAV increases, the production and secretion of progranulin are promoted, thereby alleviating inflammation via TNFRs.^18^

We estimated the optimum cut‐off value of serum progranulin for predicting severe AAV and found two cut‐offs, one with high sensitivity and the other with high specificity. We demonstrated that the two cut‐off values showed a significant RR for the cross‐sectional severe AAV. Next, multivariable logistic regression analysis was performed to obtain an independent predictor of severe AAV, but both cut‐offs could not act as an independent predictor. This might be because of the indirect role of progranulin in negative feedback, as opposed to a direct pro‐inflammatory role. Nevertheless, we believe that this study may provide valuable information on the potential application of serum progranulin as a biomarker for detecting the cross‐sectional AAV activity and predicting the risk for severe AAV.

To the best of our knowledge, this is the first study to investigate the clinical implication of serum progranulin in AAV patients. However, the number of patients included in this study was too small to obtain generalised conclusions that could be applied to AAV patients in real clinical settings. A future study with a larger cohort of AAV patients will not only validate these results but also provide more reliable information on the clinical implications of changes in serum progranulin observed in AAV patients.

In conclusion, progranulin might play an anti‐inflammatory role in AAV pathogenesis and serum progranulin could be used as a predictive marker for high activity of AAV.

## CONFLICTS OF INTEREST

The authors declare they have no conflicts of interest.

## Supporting information

Figure S1

## Data Availability

All data generated or analysed during this study are included in this published article and its supplementary information files.

## References

[1] He Z , Bateman A . Progranulin gene expression regulates epithelial cell growth and promotes tumor growth in vivo. Cancer Res. 1999;59(13):3222‐3229. 10397269

[2] Toh H , Chitramuthu BP , Bennett HP , Bateman A . Structure, function, and mechanism of progranulin; the brain and beyond. J Mol Neurosci. 2011;45(3):538‐548. 21691802 10.1007/s12031-011-9569-4

[3] Paushter DH , Du H , Feng T , Hu F . The lysosomal function of progranulin, a guardian against neurodegeneration. Acta Neuropathol. 2018;136(1):1‐17. 29744576 10.1007/s00401-018-1861-8 PMC6117207

[4] Jian J , Li G , Hettinghouse A , Liu C . Progranulin: a key player in autoimmune diseases. Cytokine. 2018;101:48‐55. 27527809 10.1016/j.cyto.2016.08.007 PMC5303690

[5] Wang S , Wei J , Fan Y , et al. Progranulin is positively associated with intervertebral disc degeneration by interaction with IL‐10 and IL‐17 through TNF pathways. Inflammation. 2018;41(5):1852‐1863. 29992506 10.1007/s10753-018-0828-1

[6] Cui Y , Hettinghouse A , Liu CJ . Progranulin: a conductor of receptors orchestra, a chaperone of lysosomal enzymes and a therapeutic target for multiple diseases. Cytokine Growth Factor Rev. 2019;45:53‐64. 30733059 10.1016/j.cytogfr.2019.01.002 PMC6450552

[7] Kitching AR , Anders H‐J , Basu N , et al. ANCA‐associated vasculitis. Nat Rev Dis Primers. 2020;6(1):71. 32855422 10.1038/s41572-020-0204-y

[8] Jennette JC , Falk RJ . Pathogenesis of antineutrophil cytoplasmic autoantibody‐mediated disease. Nat Rev Rheumatol. 2014;10(8):463‐473. 25003769 10.1038/nrrheum.2014.103

[9] Watts R , Lane S , Hanslik T , et al. Development and validation of a consensus methodology for the classification of the ANCA‐associated vasculitides and polyarteritis nodosa for epidemiological studies. Ann Rheum Dis. 2007;66(2):222‐227. 16901958 10.1136/ard.2006.054593 PMC1798520

[10] Jennette JC , Falk RJ , Bacon PA , et al. 2012 revised international Chapel Hill consensus conference nomenclature of vasculitides. Arthritis Rheum. 2013;65(1):1‐11. 23045170 10.1002/art.37715

[11] Han CW , Lee EJ , Iwaya T , Kataoka H , Kohzuki M . Development of the Korean version of short‐form 36‐item health survey: health related QOL of healthy elderly people and elderly patients in Korea. Tohoku J Exp Med. 2004;203(3):189‐194. 15240928 10.1620/tjem.203.189

[12] Mukhtyar C , Lee R , Brown D , et al. Modification and validation of the Birmingham vasculitis activity score (version 3). Ann Rheum Dis. 2009;68(12):1827‐1832. 19054820 10.1136/ard.2008.101279

[13] Guillevin L , Pagnoux C , Seror R , et al. The Five‐Factor Score revisited: assessment of prognoses of systemic necrotizing vasculitides based on the French Vasculitis Study Group (FVSG) cohort. Medicine (Baltimore). 2011;90(1):19‐27. 21200183 10.1097/MD.0b013e318205a4c6

[14] Bhamra K , Luqmani R . Damage assessment in ANCA‐associated vasculitis. Curr Rheumatol Rep. 2012;14(6):494‐500. 22983618 10.1007/s11926-012-0291-1

[15] Tanaka A , Tsukamoto H , Mitoma H , et al. Serum progranulin levels are elevated in patients with systemic lupus erythematosus, reflecting disease activity. Arthritis Res Ther. 2012;14(6):R244. 23140401 10.1186/ar4087 PMC3674629

[16] Holle JU , Windmöller M , Lange C , Gross WL , Herlyn K , Csernok E . Toll‐like receptor TLR2 and TLR9 ligation triggers neutrophil activation in granulomatosis with polyangiitis. Rheumatology (Oxford). 2013;52(7):1183‐1189. 23407387 10.1093/rheumatology/kes415

[17] Tang W , Lu Y , Tian Q‐Y , et al. The growth factor progranulin binds to TNF receptors and is therapeutic against inflammatory arthritis in mice. Science. 2011;332(6028):478‐484. 21393509 10.1126/science.1199214 PMC3104397

[18] Hasegawa M , Nishii C , Ohashi A , et al. Expression of tumor necrosis factor receptors on granulocytes in patients with myeloperoxidase anti‐neutrophil cytoplasmic autoantibody‐associated vasculitis. Nephron Clin Pract. 2009;113(3):c222‐c233. 19690440 10.1159/000235242

[19] Yamamoto Y , Takemura M , Serrero G , et al. Increased serum GP88 (Progranulin) concentrations in rheumatoid arthritis. Inflammation. 2014;37(5):1806‐1813. 24803297 10.1007/s10753-014-9911-4

[20] Wei F , Zhang Y , Zhao W , Yu X , Liu CJ . Progranulin facilitates conversion and function of regulatory T cells under inflammatory conditions. PLoS One. 2014;9(11):e112110. 25393765 10.1371/journal.pone.0112110 PMC4230946

