# Supplementary figures and images for: Serum progranulin as a predictive marker for high activity of antineutrophil cytoplasmic antibody‐associated vasculitis
Source: J Clin Lab Anal. 2021 Oct 9;35(11):e24048. doi: 10.1002/jcla.24048 (PMC8605163; doi:10.1002/jcla.24048)

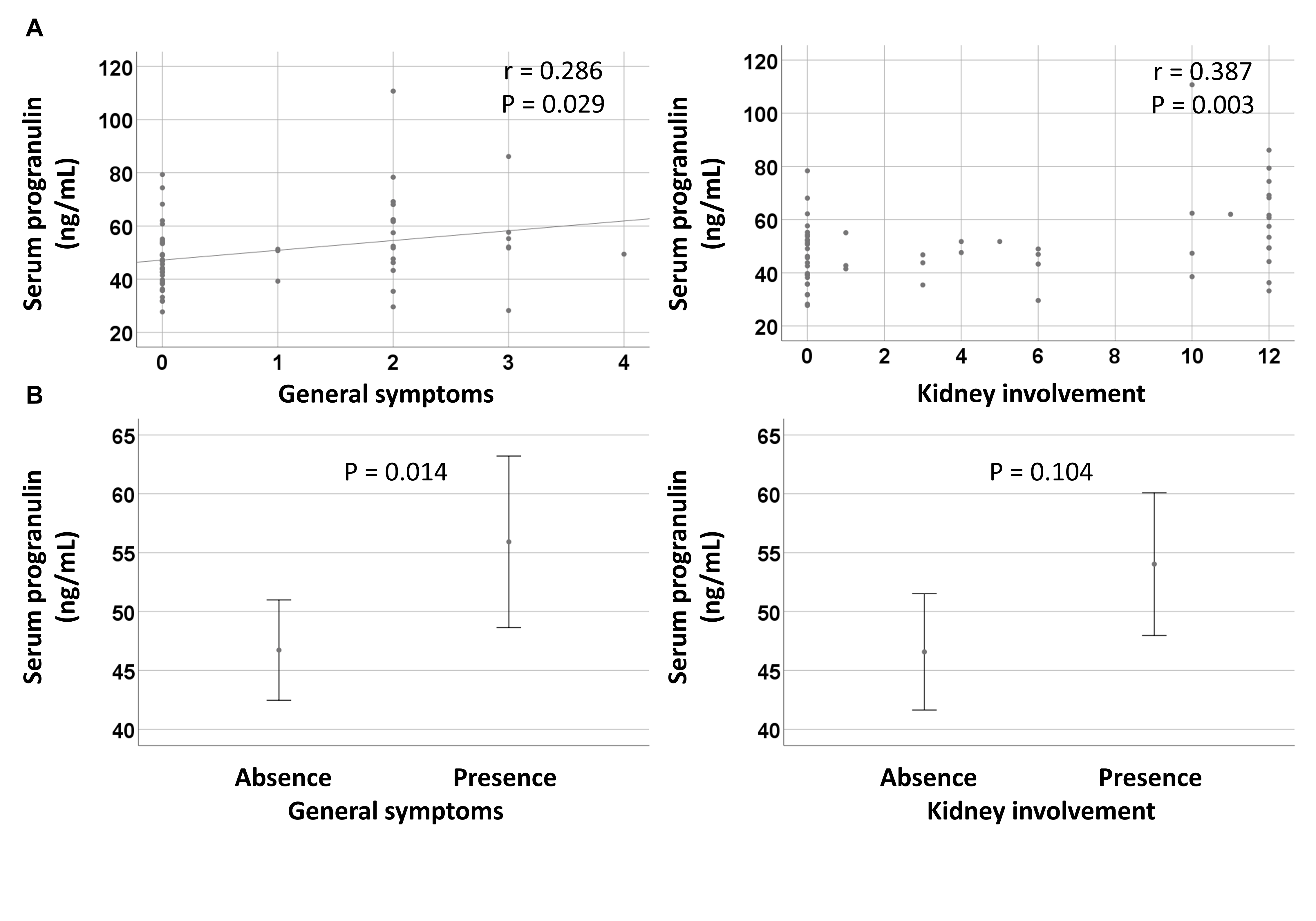

Supplement: Supplementary file 1 — Figure S1 [file JCLA-35-e24048-s001.tif]
